# Upper Extremity Stress Fractures

**DOI:** 10.1186/s40798-024-00769-7

**Published:** 2024-09-26

**Authors:** Ezekial J. Koslosky, David M. Heath, Cameron L. Atkison, Anil Dutta, Christina I. Brady

**Affiliations:** 1 Department of Orthopaedic Surgery, UT Health Science Center San Antonio, 7703 Floyd Curl Drive, San Antonio, TX 78229 USA; 2 Department of Orthopaedic Surgery, University Health System San Antonio, 4502, Medical Drive, San Antonio, TX 78229 USA

**Keywords:** Stress fracture, Clavicle, Scapula, Ribs, Humerus, Ulna

## Abstract

**Background:**

Stress injuries are often missed secondary to their insidious onset, milder symptoms, and subtle or initially absent findings when imaged.

**Main Body:**

This review aims to provide strategies for evaluating and treating upper extremity stress fractures. This article outlines the classic presentation of each fracture, the ages during which these injuries often occur, the relevant anatomy and biomechanics, and the mechanism of each injury. Diagnostic imaging and management principles are also discussed, including the use of conservative versus surgical management techniques.

**Short Conclusion:**

Upper extremity stress fractures are often mild injuries that resolve with conservative management but can lead to more serious consequences if ignored. Given their increasing incidence, familiarity with diagnosis and management of these injuries is becoming increasingly pertinent.

## Key Points

- Stress injuries are often missed secondary to their insidious onset, milder symptoms, and subtle or initially absent findings when imaged.
- This review aims to provide strategies for evaluating and treating upper extremity stress fractures.

## Background

Repetitive stress injuries are common in younger people and negatively affect athletic performance. They are caused by repetitive stress to bone over time coupled with fatigue of the supporting musculature [1]. This repetitive stress is hypothesized to cause ongoing microvascular trauma, limiting the blood supply, impairing osteogenesis, and consequently weakening the physis [2]. The microvascular trauma is compounded by an increase in local osteoclastic activity, weakening adjacent metaphyseal bone [2, 3]. Over time, this gradual weakening of the bone by repeated stress can lead to stress fractures in otherwise healthy athletes [2].

Stress injuries will affect roughly 40% of athletes at some point during their career, with 80–95% of these injuries affecting the lower extremities and only 5–20% affecting the upper extremities [4]. However, upper extremity stress fractures are becoming more common as the demand placed on young athletes and our ability to diagnose these injuries continue to increase [4].

When evaluating for stress injuries, a thorough history, sensitive physical exam, and appropriate imaging are essential. This review focuses on various clinical clues that can assist in diagnosis, which can be challenging to detect on standard radiographs alone. While radiograph sensitivity does improve with time, roughly 70% of stress fractures are not visible on initial X-rays [5]. These include inciting activities and clinical presentation of each type of injury. Early diagnosis is crucial as most stress injuries can be treated conservatively early in their clinical courses but can become debilitating, cause growth disturbances, and may require surgical intervention if allowed to progress [4].

## Clavicle

### Medial Clavicle Stress Fractures

**Fig. 1:**
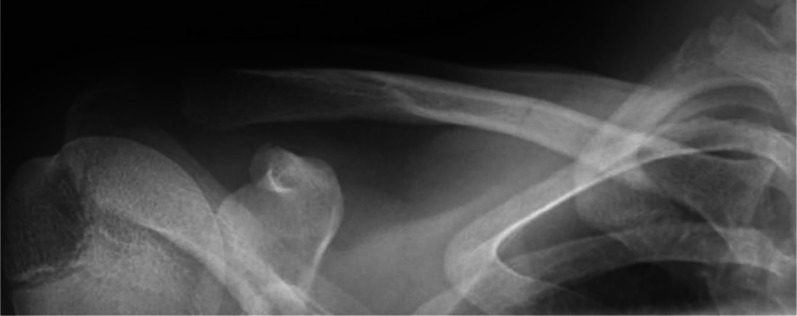
Zanca view demonstrating medial clavicle stress fracture in an adolescent weightlifter. Reproduced from Kang et al. [46], with permission

The clavicle is subject to stress from the sternocleidomastoid and trapezius pulling it superiorly and the pectoralis major and deltoid pulling it inferiorly. Usually, these forces are balanced, dissipating the force across the clavicle, but fatigue creates an imbalance that intensifies bending and torsional forces [6]. Although rare, this imbalance has led to reported cases of medial clavicle stress fractures in athletes ranging from 10 to 30 years old playing sports that require upper extremity endurance and subject the clavicle to repetitive trauma, such as rowing, throwing, diving, and gymnastics [6, 7]. Patients often present with insidious onset medial clavicular pain, tenderness to palpation, and limited shoulder abduction secondary to pain [7]. The fracture can be visualized on X-ray, shown in Fig. 1, and should be managed with 4–8 weeks of rest, a short course of physical therapy, and gradual return to activity [6, 7]. Conservative management has allowed for complete return to sport in all reported cases, usually 8 weeks post-injury [6, 7].

### Distal Clavicle Osteolysis

**Fig. 2:**
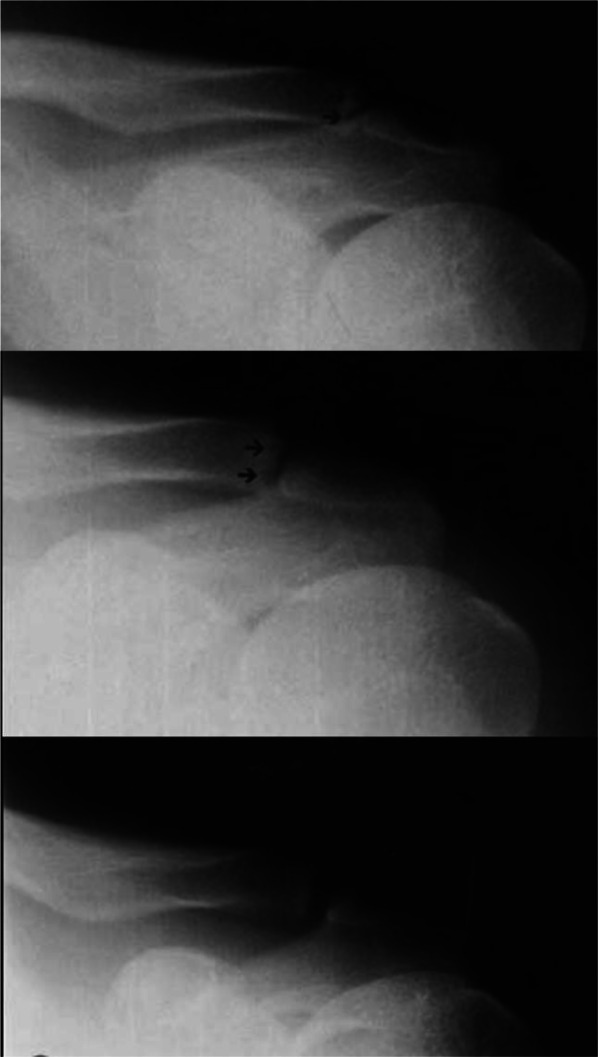
Clavicle X-rays. Top: cystic erosions noted at distal clavicle (arrows). Middle: six months later demonstrating decreased acromioclavicular joint space and reorganization of distal clavicle (arrows). Bottom: One year after initial visit, showing normal distal clavicle. Reproduced from Mestan et al. [47], with permission

Distal clavicle osteolysis can be defined as chronic erosion and resorption of the distal clavicle secondary to acromioclavicular joint microtrauma as the shoulder repeatedly hyperextends during bench press, overhead lifts, and pushing activities [8]. Stress-induced distal clavicle osteolysis classically presents as a 20–30-year-old male weightlifter with insidious onset anterior shoulder pain [8]. One retrospective study that analyzed 1432 magnetic resonance imaging (MRI) results of 13–19 year-olds with shoulder pain reported 6.5% of cases had atraumatic distal clavicle osteolysis, with 76% being male [9]. Patients report pain that worsens at night following a day of intense lifting that is relieved by rest [8]. The physical exam is often notable for acromioclavicular joint tenderness and pain with cross-body adduction [8]. Bilateral Zanca view X-rays demonstrate resorption, erosion, and cystic changes confined to the distal clavicle with concomitant acromioclavicular joint space widening (Fig. 2). However, any involvement of the acromion is suggestive of another pathology [8, 10]. Indeterminant X-rays in the setting of high clinical suspicion can be followed-up with MRI, which demonstrates hyperintensity of the distal clavicle, indicating bone marrow edema [8, 11].

In teenage athletes with distal clavicle osteolysis, 93% have been reported to respond well to conservative management [8, 9, 12]. Conversely, Cahill studied forty-six men averaging 23 years of age with distal clavicle osteolysis and only 56% improved after ceasing or changing their routine [13]. The difference in outcome based on age suggests older patients are more likely to require surgery. Nevertheless, in most cases, distal clavicle osteolysis responds well to conservative management and activity modification, including altering lifting mechanics while continuing normal activities [8, 12]. However, if symptoms persist, complete cessation from all activities that engage the pectoralis major may be required until symptoms resolve [8, 12]. For pain persisting greater than three months or athletes unwilling to alter their training regimen, an acromioclavicular joint corticosteroid injection can be given for both therapeutic and diagnostic purposes [8, 12]. Patients who temporarily experienced symptom relief following steroid injection also experienced pain relief following distal clavicle excision [8].

Twenty of the forty-six men with distal clavicle osteolysis surveyed in Cahill's study required distal clavicle resections [13]. Before surgery, advanced imaging should be performed to identify associated pathology that could be assessed with full diagnostic arthroscopy, such as rotator cuff tears and biceps tendinopathy, which have been reported in up to 81% and 22% of cases respectively [8]. This can help guide surgical approach between direct superior and subacromial approaches [8, 10, 12, 14].

## Scapula

Scapular stress fractures are a unique subset of a rare fracture location, and only a few cases have been reported [7]. Lateral border stress fractures have been reported in a professional baseball player, cricket player, and an assembly line worker hypothesized to be secondary to stress from the teres minor [7, 15, 16]. A superomedial stress fracture was reported in a jogger using handheld weights, hypothesized to be secondary to supraspinatus overuse [17]. Stress fractures at the base of the acromion have been reported in a golfer, thought to be secondary to stress from the posterior deltoid while swinging, and a football player, thought to be secondary to intensive weightlifting and blocking [18, 19]. A coracoid stress fracture was reported in a trap shooter secondary to the repetitive trauma of firing 200–1000 rounds per week [20]. Initial imaging in each case consisted of X-rays with follow-up bone scans, MRI, and/or computed tomography (CT) if needed. Each was successfully managed with two to three months of rest followed by a gradual return to activity [7].

## Ribs

Rib stress fractures often present with an insidious onset, nonspecific chest, shoulder, or back pain with movement [21]. The exam is notable for point tenderness to palpation and potentially a palpable callus over advanced fractures [22]. Stress fractures can occur at any location along any rib, but are commonly seen anterolaterally on the first rib, posterolaterally on ribs five through nine, or posteromedially on ribs two through seven [22, 23].

Stress fractures of the first rib are typically seen at the subclavian artery groove between the anterior and middle scalene muscles secondary to trauma or repetitive overhead motion, such as in throwing sports, in which the scalene muscles pull superiorly, and the serratus anterior and intercostal muscles pull inferiorly [22, 24]. These fractures are classically present as subacute to chronic shoulder, scapular, or upper back pain on the dominant side of 13 to 25-year-old athletes [24].

Stress fractures of posterolateral ribs 5–9 have been reported in golfers, gymnasts, swimmers, ballet dancers, and even patients with chronic cough [22]. However, they are most notable among 22–27-year-old elite rowers, affecting 9.2% of this population [21]. The proposed mechanism is rib cage compression, in which ribs are compressed by the anterior pull of the arms on the oar and the posterior pull of scapula-retracting muscles during a rowing stroke [21]. This compressive force is likely compounded by the external oblique and rectus abdominis muscles during forced expiration [21].

Stress fractures of posteromedial ribs 2–7 are rare and often specific to inexperienced, frequent golfers averaging 39 years of age [23, 25]. Repeatedly striking the ground with the club creates a traction force from the serratus anterior that can lead to rib fracture on the golfer's non-dominant side [23]. Classically these patients complain of vague discomfort in their upper back that is often misdiagnosed as a muscle strain [23].

Diagnosis and management of all rib stress fractures are relatively similar. All cases should initially be worked-up with X-rays. However, up to 60% of cases are missed on chest X-ray [22]. For first rib fractures, cervical spine X-rays are considered the gold standard, with a sensitivity of 97%. This is in comparison to chest X-rays with a 20% sensitivity and shoulder X-rays with 46% sensitivity [24, 26, 27].

Once diagnosed, most cases can be managed with four to six weeks of rest from aggravating activities followed by a gradual return to sport [24]. However, first rib fractures are a notable exception due to a high nonunion rate of 29% [24]. Despite this, athletes were allowed to return to sport after an average of 3.4 months if they were asymptomatic [24, 28]. Unfortunately, of the nonunion cases, 43% had a complicated course, including reports of thoracic outlet syndrome, Horner syndrome, and brachial plexus palsy [24, 28]. Development of any neurovascular symptoms following a first rib fracture should be evaluated via a CT angiogram with possible first rib resection to relieve symptoms [24].

## Humerus

Humeral stress fractures can occur secondary to activities like throwing and swinging a racquet, which places torsional stress on the humerus as it rapidly transitions from a "cocked" position through an acceleration phase to follow-through [29]. Normally, proper mechanics, leg drive, and contributions from the biceps and triceps on the humeral shaft help to dissipate the torsional stress [29]. However, fatigue gradually reduces these protective contributions by causing athletes to compromise their form to generate more power or alleviate developing pain [29]. In younger athletes with open growth plates, extending the arm too far back during the cocking phase places excess stress on the proximal humerus, which, when coupled with distraction forces, can lead to proximal humeral epiphysiolysis (Little League shoulder) [29, 30]. In spiral fractures, quickening of the acceleration phase prevents the shoulder cocking muscles from relaxing before the accelerating muscles take over, placing torsional stress across the humerus [29].

### Little League Shoulder

**Fig. 3:**
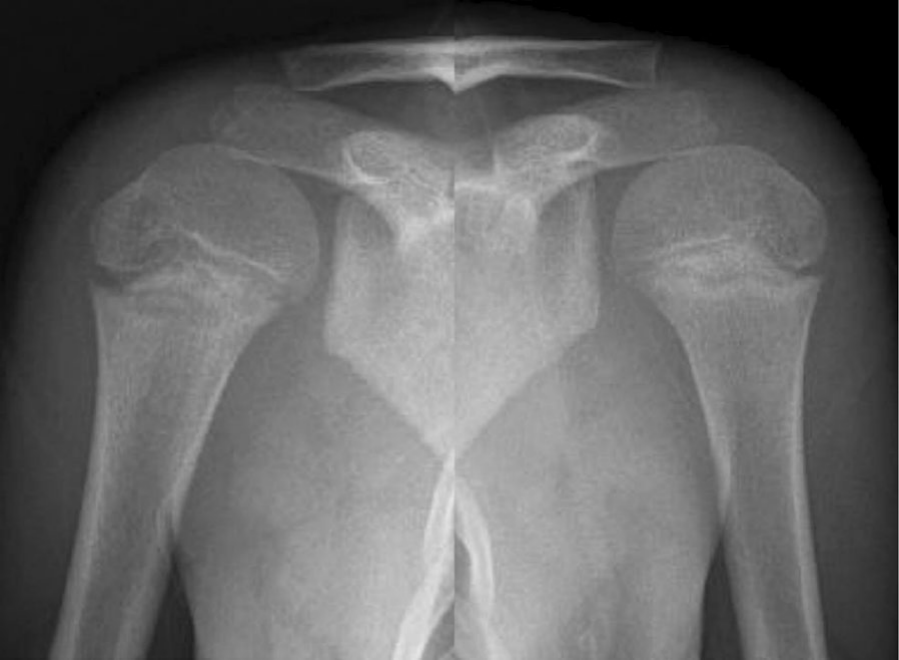
Little League shoulder. Anterior-posterior radiographs of bilateral proximal humeri of the same patient demonstrating widening of the proximal humeral physis on the right as compared with the normal left side. Reproduced from Stanley et al. [48], with permission

Little League shoulder is a Salter-Harris Type 1 torsional stress fracture of the proximal humeral physis affecting roughly 5% of all 9 to 12-year-old Little League players [31, 32]. However, this study likely underestimates the prevalence given that the classic patient is an 11–16-year-old pitcher with acute or insidious onset shoulder pain while throwing that improves with rest [30, 31]. Patients may also mention a decline in pitch accuracy and velocity, and 13% will report concomitant elbow pain [30, 31]. Little League elbow, discussed in Little League Elbow section, also develops secondary to overuse and poor mechanics in skeletally immature overhead-throwing athletes [30, 31]. On physical exam, most patients will have tenderness to palpation over their proximal-lateral humerus, and nearly half will have a painful and altered range of motion [31]. Some less common exam findings include glenohumeral internal rotation deficit (16%) and pain/weakness with resisted external rotation (10%), abduction (5%), and internal rotation (2%) [31]. Clinical suspicion is confirmed with bilateral shoulder X-rays, shown in Fig. 3, demonstrating proximal humeral physeal widening of the dominant shoulder compared to the nondominant [31]. Proximal humeral physeal widening can also be found incidentally but warrants no further action unless the patient has associated symptoms [31]. If X-rays are negative but clinical suspicion remains high, MRI can be obtained to look for proximal physeal edema [30, 31]. In a study evaluating 89 patients diagnosed with Little League shoulder, 76% of cases were confirmed with X-rays [33]. Of the patients with indeterminant X-rays, 24% underwent MRI, all of which confirmed Little League shoulder [33].

Little League shoulder is managed non-operatively, with most athletes requiring three to four months' rest from throwing while continuing non-aggravating athletic activities [31]. Following resolution of symptoms, a gradual return to sport can be attempted with potential benefit from simultaneous physical therapy, adjusting mechanics, reducing the number of breaking balls thrown, and implementing age-based pitch counts [31]. Numerous providers and institutions have published their own return to play protocols online, so providers without their own regimen can easily select one that best suits the patient’s injury and timetable. Additionally, shoulder flexibility has been associated with better outcomes, so proper stretching before activity, breaks between pitch outings, and taking one season off from throwing per year decrease the chance of symptom recurrence and complications developing [30, 31]. Complications are rare but include premature closure of the physics, humeral head osteonecrosis, and additional stress injuries such as Little League elbow [30, 31]. One hundred percent of conservatively treated athletes had complete resolution of symptoms after two to eight months [31]. Of those who obtained follow-up imaging, 84.6% (126/149) had confirmed healing [31].

### Spiral Stress Fractures

Spiral stress fractures present with insidious onset worsening mid/distal arm pain while throwing or swinging [29]. These fractures classically present in individuals over 30 years old who do not regularly exercise and are returning to sport after prolonged time off [29]. The pain usually occurs after the throwing or swinging motion and improves with rest but can progress to occur during rest [29]. Some patients report a sudden popping sensation or rapid worsening of their pain during activity, in which case they are more likely to present with swelling and ecchymosis over their mid/distal arm with extreme pain on active and passive range of motion testing [7]. Otherwise, a physical exam may only be remarkable for tenderness to palpation over the lateral mid/distal humerus possibly with pain elicited by range of motion and strength testing [29]. All patients should obtain X-rays, which may show an obvious spiral fracture or only subtle cortical hypertrophy [29]. If needed, an MRI showing medullary edema and a linear area of diminished intensity on T1 that increases in intensity on T2 can confirm the diagnosis [29].

Except for displaced fractures, spiral stress fractures are managed non-operatively with four weeks of rest from aggravating activities with or without immobilization for one week followed by a humeral fracture brace for the remaining three weeks [29]. Once symptoms have resolved, a gradual return to sport can proceed over the next four weeks while focusing on improving mechanics, strength training, and developing appropriate warm-up routines [29]. Most patients return to competitive sports after eight to 12 weeks [29].

### Transverse Stress Fractures

Rarely, frequent weightlifting can lead to humeral stress fractures [29]. Bench press is most often implicated, but all overhead lifts place stress on the humerus as it moves from an extended to a flexed position with little to no rotation [29]. Normally, force from shoulder flexors, most notably the pectoralis major, is countered by shoulder extensors like the deltoid and rotator cuff [29]. This antagonism shifts some of the stress from the humerus to the supporting skeleton in a process known as stress shielding [29]. However, muscle fatigue impairs stress shielding and increases the bending load on the humerus, putting routine weightlifters at risk for transverse stress fractures [29].

Presentation is classically a 20–40-year-old bodybuilder or competitive weightlifter complaining of anterior proximal or mid-arm pain for the past few weeks that is aggravated by lifting [29]. The physical exam is notable for tenderness to palpation between the insertion sites of the pectoralis major and anterior deltoid, potentially with mild pain during resisted shoulder internal rotation and abduction [29]. X-rays show a transverse radiolucency with surrounding periosteal reaction and sclerosis. Management includes six to eight weeks of rest from all upper extremity lifts followed by a gradual return to activity [29]. Bracing and immobilization are not usually necessary, and all patients should ultimately return to baseline by four months post-injury [29].

### Little League Elbow

**Fig. 4:**
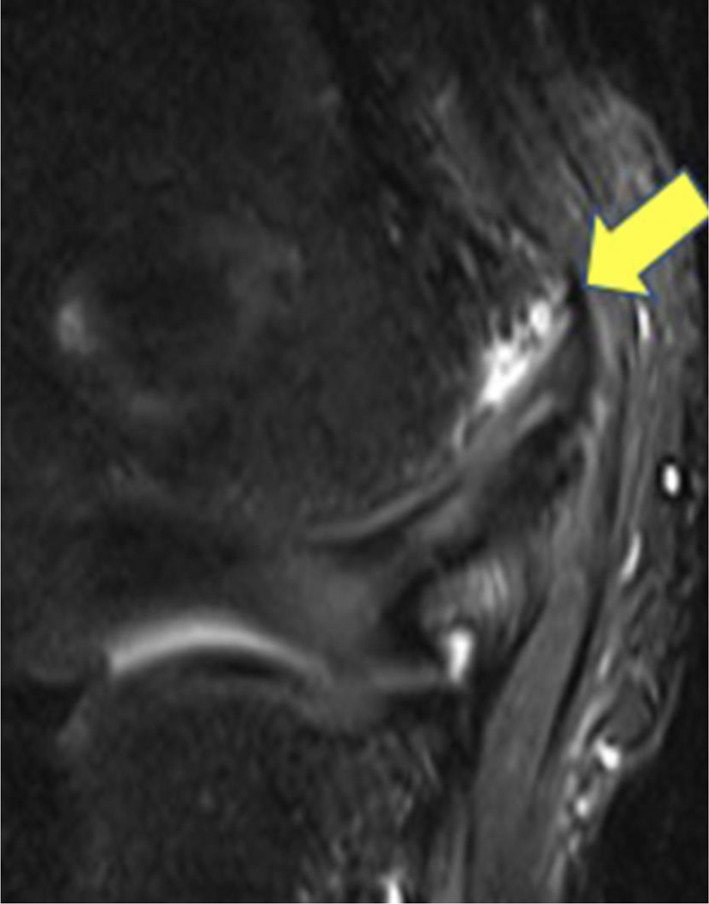
Little League elbow. T2 magnetic resonance image (MRI) demonstrating increased signal intensity at the medial epicondyle apophysis. Reproduced from Kajiwara et al. [49], with permission

Classic Little Leaguer's elbow refers to medial epicondylar apophysitis in skeletally immature athletes due to maladaptive, repetitive microtrauma to the elbow [34]. Risk factors include throwing eighty or more pitches per game, eight or more months of competitive pitching per year, and continued play despite arm pain [34]. Patients typically complain of an insidious onset of pain in the medial epicondyle that is often reproduced on physical exam, and ulnar nerve function should be assessed due to its anatomic proximity [35]. X-rays demonstrating widening or distal displacement of the medial epicondyle in comparison to the contralateral film can aid in the diagnosis, but radiographs are unremarkable in 85% of cases [36]. As shown in Fig. 4, MRI may be able to identify more subtle avulsions and early edema in the ulnar collateral ligament (UCL) [36]. Once diagnosed, complete rest from pitching for four to six weeks is indicated [36]. In patients with a severely abnormal range of motion of the elbow, an elbow brace may be prescribed [36]. After initial rest from throwing, athletes may slowly return to play with an interval throwing program for six to eight weeks while monitoring for symptoms, with cessation of throwing for several days if symptoms return [36]. Most athletes will return to full competitive play in 12 weeks [36]. Coaches and parents should be educated on early removal from play if an athlete complains of medial epicondyle pain to prevent increasing severity and complications such as ulnar nerve neuropathy, loss of motion, and inability to return to the same competitive level of play [36–38]. Although most cases of Little League elbow are treated non-operatively, medial epicondyle avulsion fractures with at least 10 mm displacement, complete UCL tears, or partial UCL tears which have failed conservative management may require surgery [37, 38]. Ultimately, prevention of Little League elbow is becoming increasingly important, with recommendations including proper stretching, avoiding breaking pitches until 13 years old, practicing proper mechanics, and participating in other activities to avoid overuse [34].

## Ulna

### Olecranon Stress Fractures

Fifty-eight percent of all upper extremity stress fractures affect the olecranon [39]. Olecranon stress fractures occur due to rapid and repetitive valgus extension, usually demanded in the rapid pitching/throwing motion [39]. The anterior band of the ulnar collateral ligament and the radius are the primary stabilizers of the valgus stress placed on the elbow [39]. Pitching requires rapid elbow extension that forces the tip of the olecranon into the olecranon fossa. The ulnar collateral ligament develops increased laxity over time due to a compensatory increase in compression on the medial aspect of the olecranon–olecranon fossa articulation [39]. Excessive tensile forces of the triceps on the olecranon also exacerbate this process which is why baseball players, javelin throwers, wrestlers, and young gymnasts are most at risk [39]. This set of pathological findings is also known as valgus extension overload syndrome [39]. Other factors contributing to this excessive load include truncal rotation, reduced shoulder external rotation, and increased elbow flexion [39].

**Fig. 5:**
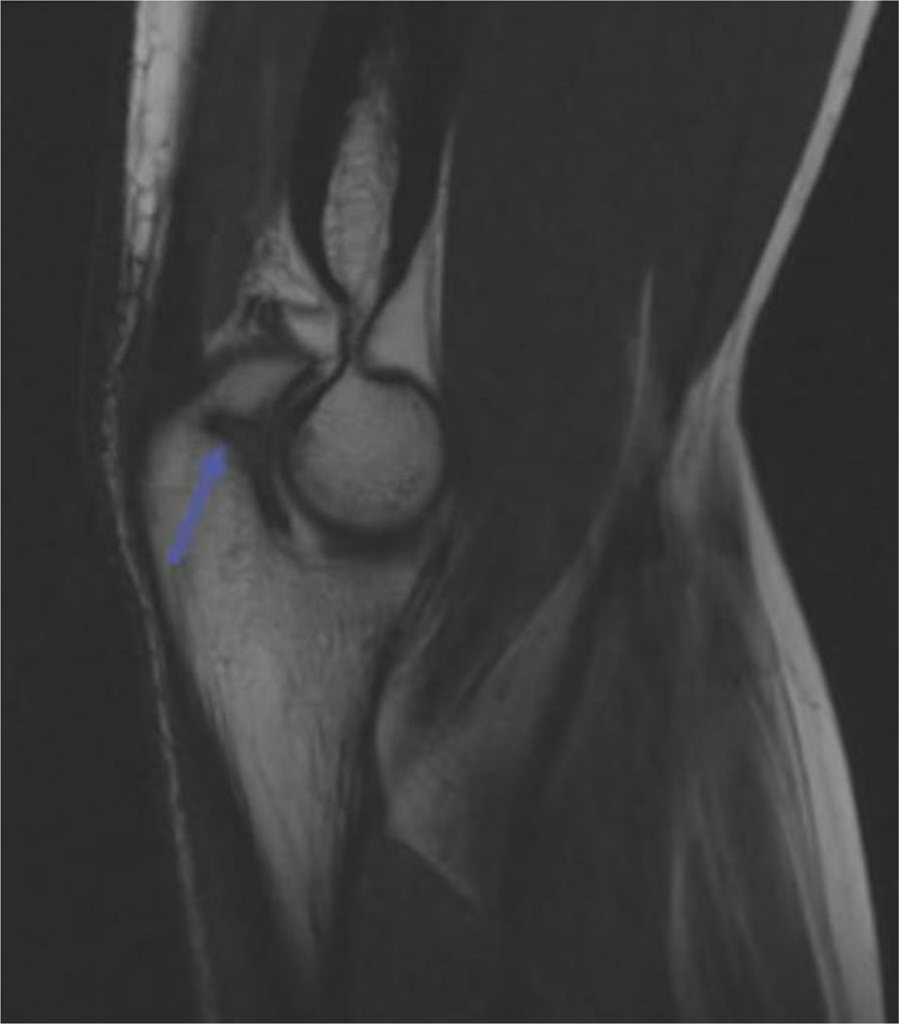
Magnetic resonance image (MRI) demonstrating olecranon stress fracture. Reproduced from Greif et al. [39], with permission

Olecranon stress fracture classification is directly related to the average age of the patient: physeal (14.1 years), transitional (16.9 years), sclerotic (18.0 years), classic (18.6 years), and distal (19.6 years) [40]. The various types of olecranon stress fractures have a similar mechanism of action, but stress different parts of the olecranon based on the maturity of bone development [40]. Physeal olecranon stress fractures occur due to extensor overload of the triceps, causing strain on the weakest portion of the olecranon, the physis [40]. X-rays may demonstrate a widened olecranon epiphyseal plate consistent with a Salter-Harris Type I fracture, and MRI, shown in Fig. 5, will demonstrate bone edema and can identify associated UCL injuries [6, 39]. If repetitive valgus forces continue, physeal olecranon stress fractures can turn into transitional stress fractures, a middle step between physeal and classic olecranon stress fractures. A classic olecranon stress fracture will show a fracture line that originates from the proximal-ulnar side and extends to the distal-radial side. On the sagittal view, the fracture line may originate from the olecranon articular surface and cross dorsally, and an MRI can be ordered if further confirmation is needed [40]. Sclerotic olecranon stress fractures are less painful and can be incidentally detected on MRI showing an extensive low-intensity area on the olecranon articular surface when UCL injury is suspected. Distal olecranon fractures classically present in adults, with X-rays demonstrating a fracture line that originates more distally than the fracture of the classical type on the cortical notch of the trochlear groove [40]. In addition, a superimposed or precipitating UCL injury and medial epicondyle avulsion fractures were found in 71%-95% of cases dependent on olecranon stress fracture type, with injury to the UCL being most associated with the transitional and classic fracture types [40].

Patients often present with increasing pain and tenderness over the olecranon process, but X-rays may be negative [6]. The high variability of radiographic findings warrants physicians being especially suspicious of an olecranon stress fracture in throwing athletes and they should have a low threshold to obtain advanced imaging and prohibit play when a stress fracture is suspected. Most olecranon stress fractures are treated with rest from the offending activity for at least eight weeks or until asymptomatic [41]. However, nonunion, an associated ulnar collateral ligament tear, or failed conservative therapy for three or more months may warrant surgery [41]. Following two weeks of immobilization, four to six weeks of recovering range of motion, and a gradual return to play, athletes were able to make a full recovery by four months post-operatively [41].

### Ulnar Shaft Stress Fractures

**Fig. 6:**
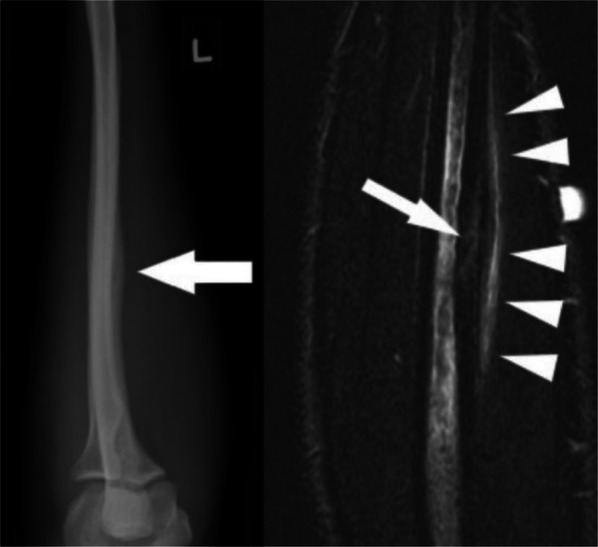
Ulnar stress fracture. (Left) Lateral radiograph of a left forearm showing cortical thickening along the volar cortex; (Right) Sagittal inversion recovery image of a forearm demonstrating periosteal, cortical and medullary edema (arrowheads) along the volar aspect of the ulnar diaphysis as well as a subtle oblique low signal fracture line (arrow). Reproduced from Dean et al. [51], with permission

Ulnar shaft stress fractures are much less common, classically presenting in 13–16 year old female athletes who perform the repetitive supination to pronation movements required by activities like softball, tennis, golf, and twirling [42–45]. Patients typically complain of insidious onset forearm pain during activity that is reproduced with palpation, pronation, and supination during the physical exam [45]. These stress fractures are not always seen in radiographs, so an MRI, shown in Fig. 6, is recommended if clinical suspicion remains despite negative X-rays [42, 45]. First-line treatment is cessation of the aggravating activity for six to eight weeks, after which nearly all athletes make a full recovery [42, 45].

## Conclusion

Upper extremity stress fractures are becoming more common as the workload placed on young athletes continues to increase. Most stress fractures resolve with conservative management but can cause unrelenting pain, loss of function, and permanent damage if ignored. Therefore, familiarity with the biomechanics, typical presentation, work-up, and treatment of each stress fracture is an important educational topic for non-orthopedic physicians, to whom many patients initially present.

## Data Availability

No data sets were generated or utilized.

## Abbreviations

MRI: Magnetic resonance imaging
CT: Computed tomography
UCL: Ulnar collateral ligament
